# Analysis of the Correlation Between Cuproptosis and Instability of Atherosclerotic Plaques

**DOI:** 10.3390/biomedicines13122983

**Published:** 2025-12-04

**Authors:** Muheremu Muhetaer, Tianwen He, Haoyan Zhu, Jiahe Wu, Jingjing Wan, Tong Zhang, Yushuang Hu, Zhibing Lu, Huanhuan Cai

**Affiliations:** 1 Department of Cardiology, Zhongnan Hospital of Wuhan University, Wuhan 430071, China; 2021283030105@whu.edu.cn (M.M.);; 2 Institute of Myocardial Injury and Repair, Wuhan University, Wuhan 430071, China; 3 Hubei Provincial Clinical Research Center for Cardiovascular Intervention, Wuhan 430071, China; 4 Department of Cardiology, Renmin Hospital of Wuhan University, Wuhan 430061, China

**Keywords:** cuproptosis, unstable atherosclerosis plaque, immune infiltration, machine learning, cluster analysis

## Abstract

**Background/Objectives**: Cuproptosis, a newly discovered form of programmed cell death, is dependent on the regulation of copper ions. The roles and mechanisms of cuproptosis-related genes (CRGs) in the instability of atherosclerotic plaques are still unclear. **Methods**: GEO microarray datasets were downloaded to analyze stable and unstable human carotid artery plaques. Differential expression analysis was performed to screen for CRGs from the Molecular Signatures Database (MSigDB). Machine learning was applied to identify key genes and cluster unstable plaque genes. The identified genes were verified by immunohistochemistry (IHC) of human carotid plaque samples, and the effect of ATOX1 on cuproptosis was detected in human umbilical vein endothelial cells (HUVEC). **Results**: This study identified 27 CRGs differentially expressed between stable and unstable plaques. Five characteristic genes (LC3A, ATP7B, ATOX1, CTR1, and NLRP3) were selected by machine learning. A diagnostic model for unstable plaques was developed based on these genes. The expression of ATOX1 and NLRP3 was increased, while LC3A and ATP7B were decreased in unstable plaques. However, there was no significant change in CTR1. The Cell Counting Kit-8 (CCK-8) assay indicated that inhibiting ATOX1 reduced CuSO_4_-induced HUVEC death. **Conclusions**: CRGs appear to influence atherosclerotic plaque formation. Five key genes (LC3A, ATP7B, ATOX1, CTR1, NLRP3) were identified as being differentially expressed in unstable plaques. Cluster analysis uncovered two subtypes (C1, C2) linked to cuproptosis and immune infiltration in unstable plaques. These genes likely affect atherosclerosis progression by influencing immune cell infiltration, thus impacting plaque stability. Furthermore, the cuproptosis-related gene ATOX1 can regulate CuSO_4_-induced HUVEC death. This study contributes to predicting plaque instability and offers potential diagnostic and therapeutic targets.

## 1. Introduction

Atherosclerosis (As) is the leading cause of cardiovascular disease (CVD) and also a chronic inflammatory disease of the artery wall caused by the increase of low-density lipoprotein level [1]. As atherogenic lipoproteins and plasma molecules infiltrate the subendothelial space through the damaged endothelium, atherosclerotic plaques can form in the vascular lumen. These advanced atherosclerotic plaques have higher instability, which may rupture and then lead to fatal diseases such as myocardial infarction and ischemic stroke [2]. It is currently believed that the immune inflammatory response is widely involved in the formation and progression of plaques, and the infiltration of various immune cells and the release of various pro-inflammatory factors can cause sustained local inflammation and oxidative stress [3]. Macrophages absorb lipids, such as oxidized low-density lipoprotein and other atherogenic lipoproteins, transforming into foam cells to form fatty stripes and initiating early atherosclerosis [4,5]. Th1 cells release tumor necrosis factor (TNF-α) and interferon-γ (IFN-γ), which play a role in promoting atherosclerosis [2]. The activation of these chemotactic inflammatory cells leads to an increase in the volume of the necrotic core, promotes the thinning and remodeling of the fibrous cap, and damages vasodilation ability, making the atherosclerotic plaque more vulnerable to rupture. In addition, cell death, such as the death of foam cells (especially programmed necrosis), also directly leads to the expansion of necrotic cores and instability of plaques [6,7]. However, its specific mechanisms remain unclear.

Recent studies have shown that cuproptosis is a new type of programmed cell death that relies on mitochondrial respiration in cells, and its mechanism is different from cell apoptosis, pyroptosis, and ferroptosis [8]. As a co-factor for various enzymes in organisms, copper participates in many physiological pathways and is related to important biological processes, including angiogenesis, response to hypoxia, and neural regulation [9]. Under normal conditions, the intracellular copper concentration remains very low [10]. When the steady-state imbalance of copper ions in cells occurs and the concentration of copper ions exceeds the threshold, copper directly binds to the lipid-acylated components of the tricarboxylic acid (TCA) cycle. The aggregation of lipid-acylated proteins, loss of Fe-S cluster proteins, and induction of HSP70 ultimately lead to acute protein toxicity stress and cell death responses [8]. As a newly discovered form of programmed cell death, cuproptosis involves various cells controlled by mitochondrial respiration. In addition, copper is closely related to the immune system [11]. One study has shown (or showed) that copper supplements can inhibit atherosclerosis by reducing the concentration of cholesterol and phospholipids in diseased tissues and narrowing atherosclerosis [12]. However, the mechanism of the influence of cuproptosis on atherosclerotic plaque instability is still unclear.

In our research, we conducted a comprehensive analysis of existing atherosclerotic plaque data and collected cuproptosis-related genes (CRGs) through bioinformatics methods to explore the role and potential mechanism of CRGs in plaque instability. At the same time, unstable plaques were clustered and classified further to explore the influence of immune infiltration and other factors. This will facilitate the early detection of unstable plaques and the prevention or treatment of plaque progression and rupture.

## 2. Materials and Methods

### 2.1. Data Resource and Preprocessing

The Gene Expression Omnibus (GEO) database (https://www.ncbi.nlm.nih.gov/ (accessed on 15 December 2023)) was used to download the atherosclerotic plaque-related microarray datasets GSE41571, GSE163154, GSE43292, and GSE28829. These four datasets were annotated separately. Then, the first three datasets (GSE41571, GSE163154, and GSE43292) were combined into a training set. The data in GSE28829 were used as a validation set. Among them, GSE41571 comes from the GPL570 platform ([HG-U133_Plus_2] Affymetrix Human Genome U133 Plus 2.0 Array), including 6 stable and 5 unstable plaques. GSE163154 comes from the GPL6104 platform (Illumina humanRef-8 v2.0 expression bead chip), including 16 stable and 26 unstable plaques. GSE43292 comes from the GPL6244 platform ([HuGene-1_0-st] Affymetrix Human Gene 1.0 ST Array [transcript (gene) version]), including 32 complete plaques and 32 advanced plaques. GSE28829 is from the GP570 platform ([HG-U133_Plus_2] Affymetrix Human Genome U133 Plus 2.0 Array), including 13 early and 16 advanced plaques. All samples were from human carotid plaques. A total of 54 CRGs were collected from previous articles [8] and the Molecular Signature Database (MsigDB) v7.0 database (http://www.gsea-msigdb.org/gsea/msigdb/ (accessed on 15 December 2023)) for subsequent bioinformatics analysis. Batch effects among datasets in the training set were eliminated through the “ComBat” function in the “SVA” package [13]. Principal Component Analysis (PCA) was also used to visualize data to observe the results of batch effect removal.

### 2.2. Differential Expression Analysis of CRGs

The combined data were analyzed using the “limma” package [14]. Differentially expressed genes (DEGs) were obtained by analyzing 54 samples from the stable plaque group and 63 samples from the unstable plaque group. A *p*-value < 0.05 was set as the cut-off standard to screen differentially expressed genes. Differentially expressed cuproptosis-related genes (DECRGs) were obtained by intersecting DEGs and CRGs for further analysis. The results were displayed as a box plot and heat map by using the R package 4.2.1 “ggplot” and “pheatmap”.

### 2.3. Functional and Pathway Enrichment Analysis

The “cluster profiler” package [15] was used to perform Gene Ontology (GO) analysis and Kyoto Encyclopedia of Genes and Genomes (KEGG) pathway enrichment analysis on these genes. *p* < 0.05 was considered statistically significant.

### 2.4. Screening of Cuproptosis-Related Key Genes

Three machine learning algorithms, including the LASSO algorithm, support vector machine algorithm, and random forest algorithm, were used to analyze DECRGs to screen for key cuproptosis-related genes associated with the instability of atherosclerotic plaque. The least absolute shrinkage and selection operator [16,17] (LASSO) is a suitable dimensionality reduction method for high-dimensional data. The LASSO model was constructed through the “glmnet” package [18], and the data was cross-validated ten times with penalty parameters to screen feature genes. The support vector machine-recursive feature elimination [19] (SVM-RFE) algorithm was used to select relevant features and remove redundant features. After training samples through the model, each feature was scored and sorted to remove the feature with the minimum feature score. Then, the model was trained again with the remaining features for the next iteration. A total of 19 cross-verifications were conducted to screen feature genes. The random forest [20] (RF) algorithm is a machine learning algorithm that can process input samples of high-dimensional features. It evaluated the importance of each feature in classification, evaluated the prediction effect through 10-time cross-validation, and determined the gene with a relative importance of >2 as the feature gene. Finally, the intersection genes of the three algorithms were used as key genes to build diagnostic models. The receiver operating characteristic (ROC) curves and the area under the curve (AUC) were used to evaluate the diagnostic effect in the training set and verification set.

### 2.5. Consensus Clustering Analysis

The “consensus clusterplus” package [21] was used for clustering analysis based on cuproptosis-related key genes in stable atherosclerotic plaque. The best clustering number was determined by integrating the results of the consistency matrix, the consistency cumulative distribution function (CDF), the area under the CDF curve, and the tracking chart. The box plot and heat map showed the difference of expression of cuproptosis-related key genes among different subtypes, and a PCA diagram was used to verify the results of the samples after typing.

### 2.6. Gene Set Variation Analysis

Gene set variation analysis [22] (GSVA) is a non-parametric and unsupervised analytical method. The “GSVA” package was used to evaluate the different KEGG pathways enriched between samples of different subtypes. The “limma” package was used to compare the GSVA score difference of different pathways among subtypes, and the results are displayed in a histogram.

### 2.7. Immune Infiltration Analysis

Cell type identification by estimating relative subsets of RNA transcripts (CIBERSORT) [23] was used to quantify the infiltration degree of 22 kinds of immune cells based on gene expression data, and the infiltration results between stable plaque and unstable plaque groups are displayed through the “vioplot” package. The histogram made by the “barplot” function of the “ggpubr” package showed the percentage of immune cells in different samples. The “ggplot” package was used to draw the correlation map between key genes and immune cells. The classification of macrophage phenotypes was based on the LM22 signature matrix used by CIBERSORT. Specifically, monocytes are characterized by the expression of CD14 and FCGR3A (CD16); M0 macrophages (undifferentiated) are identified by CD68 expression and the lack of specific M1/M2 polarization markers; M1 macrophages (pro-inflammatory) are distinguished by CD80, CD86, and NOS2; and M2 macrophages (anti-inflammatory) are characterized by MRC1 (CD206), CD163, and ARG1.

### 2.8. Construction of Nomographs

The “rms” package was used to establish a nomogram based on key genes. The calibration curve was created to evaluate the accuracy of the nomogram. Decision curve analysis was used to evaluate the clinical usefulness of the nomograph.

### 2.9. Patients and Controls

In this study, 9 atherosclerotic plaque samples from patients undergoing endarterectomy in Zhongnan Hospital of Wuhan University were collected. All patients underwent preoperative MRI or CTA to evaluate the condition of the carotid artery, and each carotid artery lesion was classified using a modified American Heart Association (AHA) MRI or CTA-recognized carotid plaque classification. We classified IV–V and VI types as unstable plaques and other types as stable plaques. All preoperative CTA or MRI images were independently analyzed by two experienced researchers, who were unaware of the clinical manifestations and identity of each patient. Any differences were discussed and resolved by two investigators. This study was approved by the Medical Ethics Committee of Central South Hospital of Wuhan University. All patients and their families included in the study were informed of this study and signed informed consent forms. This study meets the standards established by the Helsinki Declaration.

### 2.10. Histological Analysis and Immunohistochemistry (IHC) Analysis

The carotid atherosclerotic plaque was removed using standard surgical techniques. The plaque specimen was immediately taken to the laboratory and stored in a fresh 4% paraformaldehyde solution at 4 °C. Afterwards, it was fixed with 10% buffered formalin and embedded in paraffin blocks, and 4 μm thick sections were prepared for subsequent staining. Subsequently, the slices were placed in a 3% hydrogen peroxide solution for 10 min to quench endogenous peroxidase activity. Then, 3% BSA (Wuhan Saiwei Biotechnology Co., Ltd., Wuhan, China) serum was dropped into the histochemical circle for blocking and stained with antibodies corresponding to the key genes. When using freshly prepared DAB color solution (Wuhan Saiwei Biotechnology Co., Ltd., Wuhan, China) and controlling the color development time under a microscope, the positive result is brownish yellow, and the color development is terminated by rinsing the slices with tap water. After about 3 min of re-staining with hematoxylin, the hematoxylin blue solution returned to blue. Finally, we dehydrated the slices and sealed them transparently. We interpreted the results under a white light microscope. The final IHC staining scores were assessed by two experienced pathologists working independently (i.e., unaware of each other’s results) and without knowledge of the clinical grouping information for the samples (blinded). The staining intensity score was 0 (no staining), 1 (light yellow staining), 2 (yellow staining), and 3 (deep yellow staining). Staining area: 0 points (<5%), 1 point (5%~25%), 2 points (25%~50%), 3 points (50%~75%), and 4 points (>75%). Immunohistochemical staining was used to calculate the final score using Image J 1.8.0, and statistical analysis was performed using Graphpad 9.

### 2.11. CCK-8 Cytotoxicity Assay

HUVEC cells in good growth status were used to prepare cell suspensions and counted. They were seeded into 96-well plates at approximately 100 μL per well, with three technical replicates per treatment condition. All HUVEC experiments were performed with at least three independent biological replicates. In each independent experiment, untreated cells served as the negative control, alongside the corresponding solvent control (e.g., DMSO solvent control for DC-AC50 inhibitors). CCK-8 assay results are presented as the mean ± standard deviation (SD) of three independent biological replicates. Different concentrations of CuSO_4_ were added according to the experimental design. The 96-well plate was placed in the incubator and incubate for 24 h (37 °C, 5% CO_2_). After the end of culture, 10 μL of CCK8 detection solution was added to each well in a 96-well plate and then placed in an incubator to incubate in the dark for 1–2 h. Absorbance (OD) was measured at 450 nm using a microplate reader after incubation was complete. The result calculation was as follows: cell survival rate = [(experimental well − blank well)/(control well − blank well)] × 100% cell inhibition rate = [(control well − experimental well)/(control well − blank well)] × 100%

### 2.12. Statistical Analysis

All bioinformatics and statistical analyses were performed using R language (version 4.2.1) and GraphPad Prism (version 9.0), and flow cytometry data were mainly analyzed using CytExpert 2.3 and FlowJo V10. A Wilcoxon test was used to estimate expression differences between the two groups. Correlations between variables were determined using Pearson correlation tests. Differences were considered statistically significant if *p* < 0.05.

## 3. Results

### 3.1. Differential Expression Analysis in Unstable and Stable Carotid Artery Plaques

**Figure 1:**
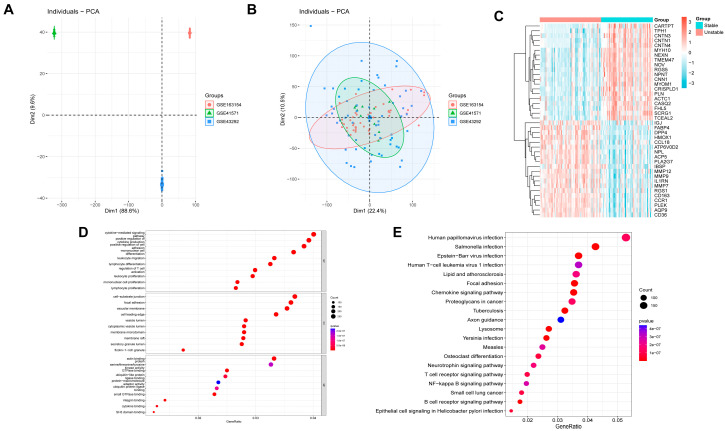
Differential expression analysis and enrichment analysis in the combined dataset of GSE163154, GSE41571 and GSE43292. (**A**) The PCA plot shows the combined data set before the batch effect. (**B**) The PCA plot shows the combined data set after the batch effect. (**C**) The heat map shows the differential expression genes between stable and unstable atherosclerotic plaque. (**D**,**E**) The bubble diagrams show GO annotation and KEGG pathway enrichment analysis for differentially expressed genes.

The atherosclerotic plaque-related datasets GSE163154, GSE41571 and GSE43292 were downloaded from the Gene Expression Omnibus (GEO) database, including 63 patients with unstable carotid atherosclerosis and 54 patients with stable carotid atherosclerosis. The three datasets were combined, and the batch effect was removed for variance analysis (Figure 1A,B). The heat map showed the up- and down-regulated genes in the combined dataset, with a total of 7946 differentially expressed genes (Figure 1C). GO annotation and KEGG enrichment analysis showed that these genes were enriched in functions and pathways related to immune regulation, such as “positive regulation of cell adhesion”, “leukocyte migration”, “chemokine signaling pathway”, “B cell receptor signaling pathway” and other pathways (Figure 1D,E).

### 3.2. Differential Expression Analysis of CRGs

**Figure 2:**
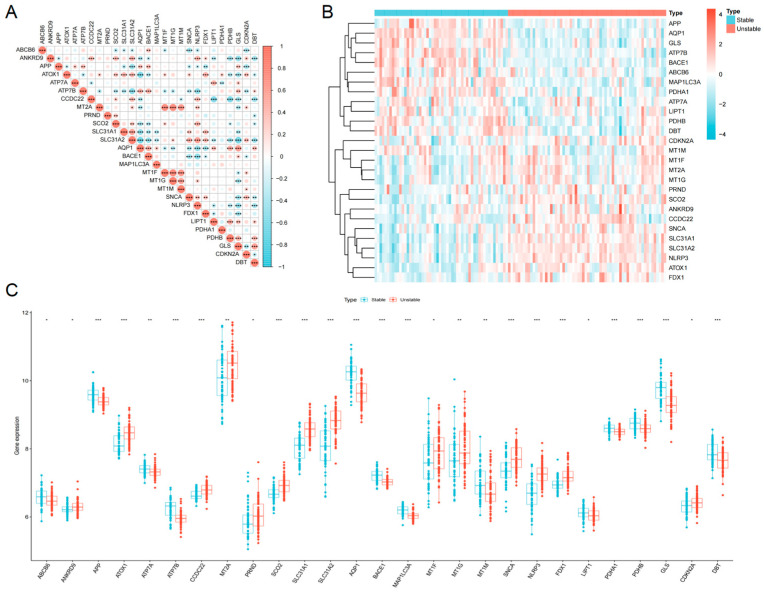
Identification of CRGs differentially expressed between stable and unstable atherosclerotic plaques. (**A**) The scatter diagram shows the correlation between these differentially expressed CRGs. (**B**) The heat map shows the expression pattern of differentially expressed CRGs in different samples. (**C**) The box plot shows all differentially expressed CRGs. (**A**,**B**) * *p* < 0.05; ** *p* < 0.01; *** *p* < 0.001.

To investigate the specific role of cuproptosis, we intersected these DEGs with 54 known CRGs. This screening yielded 27 differentially expressed cuproptosis-related genes (DECRGs). Among these, 14 genes, including the key cuproptosis regulator FDX1, were up-regulated in the unstable plaque group, while 13 genes were down-regulated (Figure 2B,C). A correlation matrix revealed complex inter-relationships between these 27 DECRGs (Figure 2A).

### 3.3. Functional Enrichment Analysis of DECRGs

**Figure 3:**
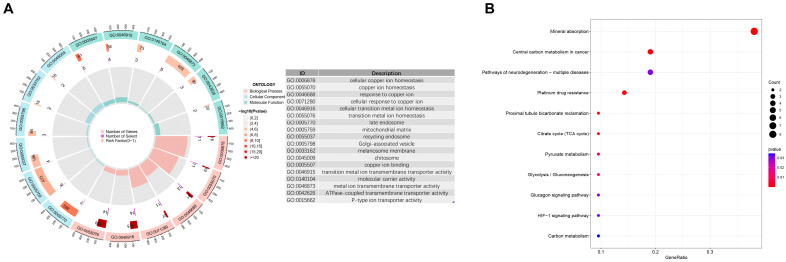
GO annotation and KEGG analysis of CRGs. (**A**) The circle diagram shows the GO annotation of these differentially expressed CRGs. (**B**) The bubble diagram shows enrichment analysis of the KEGG pathway of differentially expressed CRGs.

To elucidate the biological significance of these 27 DECRGs, we performed GO and KEGG pathway enrichment analyses. GO analysis revealed significant enrichment in terms directly related to copper metabolism (Figure 3A). The top biological process (BP) terms included “cellular copper ion homeostasis” and “response to copper ion”. Cellular component (CC) analysis pointed to the “mitochondrial matrix” and “late endosome”. Molecular function (MF) analysis was dominated by “copper ion binding” and “transition metal ion transmembrane transporter activity”.

Crucially, KEGG pathway analysis (Figure 3B) showed enrichment in “Mineral absorption” and “Citrate cycle (TCA cycle)”. This finding provides the first bioinformatic link between the DECRGs in unstable plaques and the core mechanism of cuproptosis, which is defined by copper-dependent targeting of lipoylated TCA cycle proteins.

### 3.4. Machine Learning Algorithm for Screening Cuproptosis-Related Key Genes

**Figure 4:**
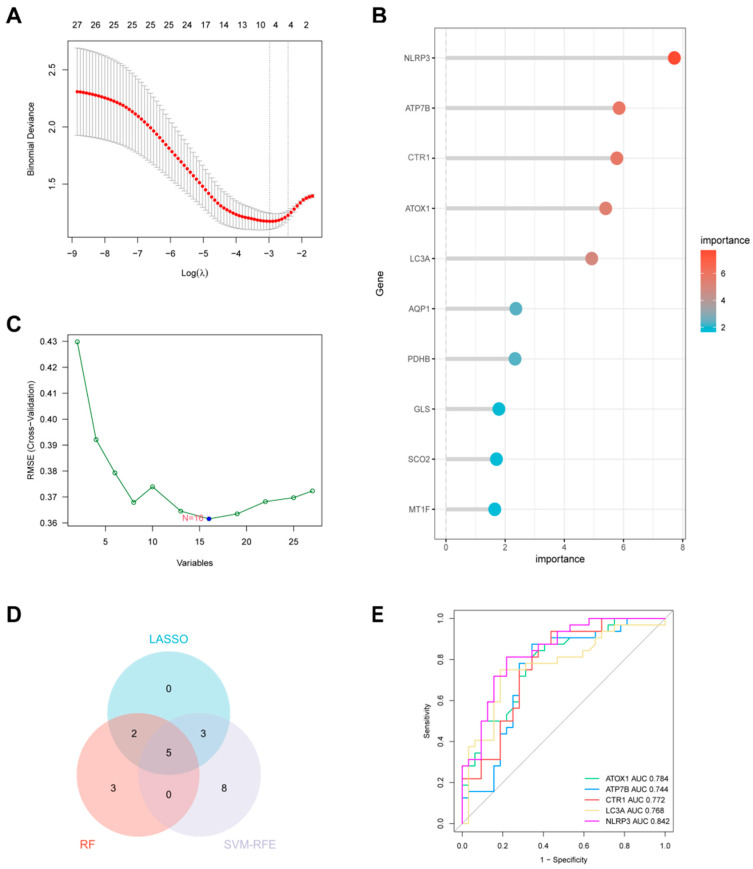
Three machine learning algorithms used to screen characteristic CRGs related to unstable atherosclerotic plaque and evaluate their diagnostic effectiveness in the combined data set. (**A**) LASSO regression analysis to determine the minimum average cross-validation error λ value. (**B**) Random forest method to determine the threshold of minimum cross-validation error. (**C**) SVM-RFE algorithm to determine the best threshold for cross-validation accuracy. (**D**) The Venn diagram shows the intersection genes of genes screened by three algorithms. (**E**) ROC curve shows the diagnostic performance of the diagnostic model based on key genes.

To identify key genes with high diagnostic potential, we applied three independent machine learning algorithms to the 27 DECRGs (Figure 4). The Least Absolute Shrinkage and Selection Operator (LASSO) regression analysis screened 10 feature genes (Figure 4A). The random forest (RF) algorithm identified 10 genes with a relative importance score > 2 (Figure 4B). The support vector machine–recursive feature elimination (SVM-RFE) algorithm identified 16 optimal feature genes (Figure 4C,D). The intersection of these three algorithms yielded a robust five-gene signature: LC3A, ATP7B, ATOX1, CTR1, and NLRP3 (Figure 4E). Among them, the expression of ATOX1, NLRP3 and CTR1 in unstable plaque increased, while the expression of ATP7B and LC3A in unstable plaque decreased (Figure 2).

### 3.5. Diagnostic Efficacy and Verification of Key Genes

**Figure 5:**
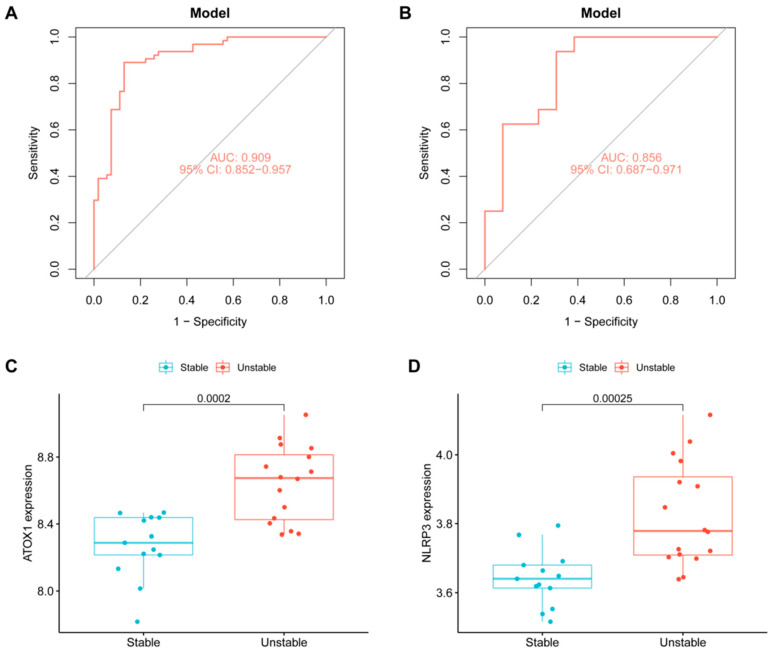
The diagnostic efficacy and expression of key genes were verified in an external dataset GSE28829. (**A**) ROC curve shows the diagnostic performance of the diagnostic model in the training set. (**B**) ROC curve shows the diagnostic performance of the diagnostic model in the validation set. (**C**) The box diagram shows the expression of ATOX1 in the validation set. (**D**) The box diagram shows the expression of NLRP3 in the validation set.

Based on the expression of these five key genes, we constructed a logistic regression diagnostic model. The model demonstrated excellent diagnostic efficacy for plaque instability in the training set, with an area under the receiver operating characteristic (ROC) curve (AUC) of 0.909 (Figure 5A). To validate this signature, we tested the model in an independent external dataset, GSE28829 (13 early vs. 16 advanced plaques). The diagnostic model performed well, achieving an AUC of 0.856 (Figure 5B). Furthermore, the expression trends of key genes were confirmed in this validation set, with ATOX1 and NLRP3 showing significantly increased expression in the advanced plaque group (Figure 5C,D). These results indicate that this five-gene signature can effectively predict atherosclerotic plaque instability.

### 3.6. Immunological Characteristics Related to Plaque Instability

Having established a diagnostic signature, we next explored its underlying biological context. Using the CIBERSORT algorithm, we analyzed the immune infiltration landscape (Figure S1). A histogram shows the infiltration proportions of 22 immune cell types across samples (Figure S1A). Violin plots reveal significant differences between stable and unstable plaques (Figure S1B).

Notably, unstable plaques exhibited higher infiltration of B cells memory, plasma cells, CD4 memory-activated T cells, gamma delta T cells, and M0 macrophages. Conversely, stable plaques showed higher infiltration of naïve B cells, CD8 T cells, CD4 memory-resting T cells, regulatory T cells (Tregs), and M2 macrophages. The significant increase in M0 macrophages (quiescent or newly recruited) and decrease in M2 macrophages (anti-inflammatory) suggest a highly inflammatory and dysregulated immune microenvironment in unstable plaques.

### 3.7. Identification of Two Novel Subtypes Within Unstable Plaques

**Figure 6:**
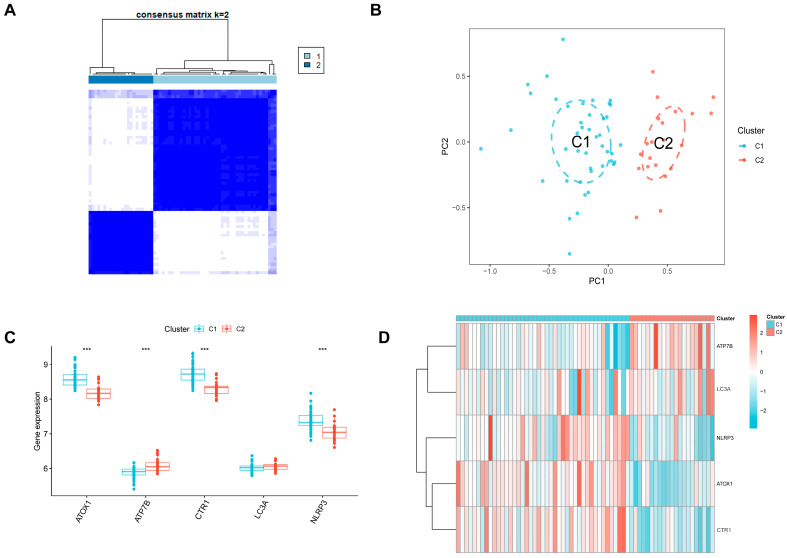
Construction of subtypes in unstable atherosclerotic plaque based on CRG-related key genes. (**A**) The heat map shows the clustering classification when the consensus matrix k = 2. (**B**) The PCA diagram shows that unstable plaque samples can be well distinguished by different subtypes. (**C**) The box diagram shows the difference in the expression of key genes among different subtypes. (**D**) The heat map shows the expression of key genes among different strains. For panel (**C**) *** *p* < 0.001.

The five-gene signature revealed significant heterogeneity. We therefore hypothesized that unstable plaques are not a monolithic entity. Based on the expression of the five key genes, we performed consensus clustering within the unstable plaque samples. The consensus matrix, CDF curve, and tracking plot all indicated that k = 2 was the optimal number of clusters (Figure 6A).

This analysis successfully stratified unstable plaques into two distinct subtypes, C1 and C2. PCA confirmed a clear separation between these two subtypes (Figure 6B). Expression analysis showed that C1 was characterized by high expression of ATOX1, NLRP3, and CTR1, while C2 was characterized by high expression of ATP7B (Figure 6C,D).

### 3.8. Biological Pathways and Immune Signatures of C1 and C2 Subtypes

**Figure 7:**
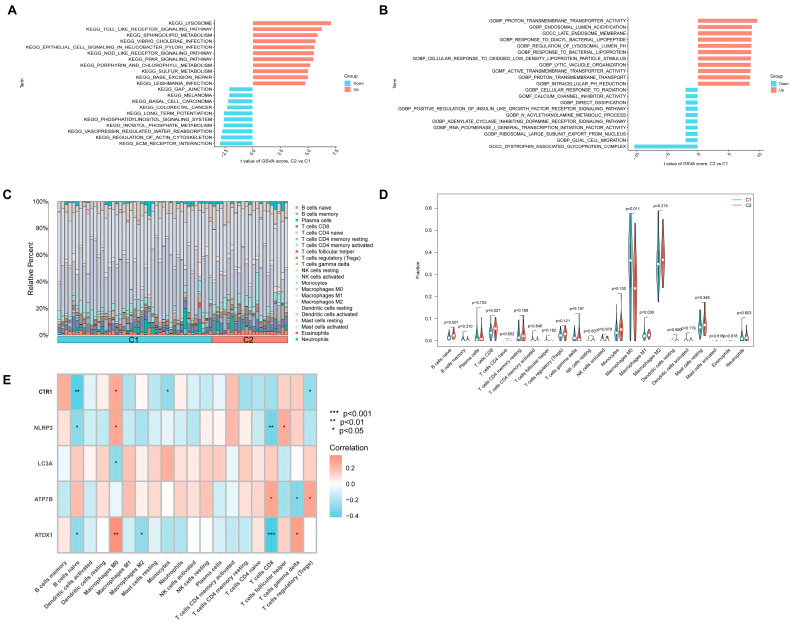
GSVA and immunocyte infiltration of two CRG-related subtypes. (**A**) GSVA shows that C1 subtype and C2 subtype were enriched to different KEGG pathways. (**B**) GSVA shows that subtype C1 and subtype C2 were annotated on different GO terms. (**C**) The histogram shows the percentage of infiltrating immune cells in samples of different subtypes. (**D**) The violin plot shows the difference of immune cell infiltration between different subtypes. (**E**) The correlation diagram shows the correlation between key genes and immune cells. For panel (**E**) * *p* < 0.05; ** *p* < 0.01; *** *p* < 0.001.

To understand the functional differences between these subtypes, we conducted Gene Set Variation Analysis (GSVA). Compared to C1, the C2 subtype showed significant up-regulation of pathways including “Toll-like receptor signaling pathway”, “Nod-like receptor signaling pathway”, “Sphingolipid metabolism”, and “PPAR signaling pathway” (Figure 7A). GO analysis confirmed C2’s enrichment in terms like “cellular response to oxidised low-density lipoprotein particle stimulus” (Figure 7B).

We then performed immune infiltration analysis on the two subtypes (Figure 7C). The results were striking: the C2 subtype exhibited a “hot” immune phenotype, with significantly higher infiltration of naive B cells, CD8T cells, and M1 macrophages (Figure 7D).

In contrast, the C1 subtype showed higher infiltration of M0 macrophages (Figure 7D). Correlation analysis (Figure 7E) further linked this finding to the key genes: the C1-defining genes (ATOX1, NLRP3, CTR1) were all significantly positively correlated with M0 macrophages and negatively correlated with naive B cells. This suggests that the high M0 infiltration observed in unstable plaques is primarily driven by the C1 (ATOX1/NLRP3-high) subtype.

### 3.9. Construction of a Nomograph for Predicting Atherosclerotic Plaque Instability Based on Key Genes

**Figure 8:**
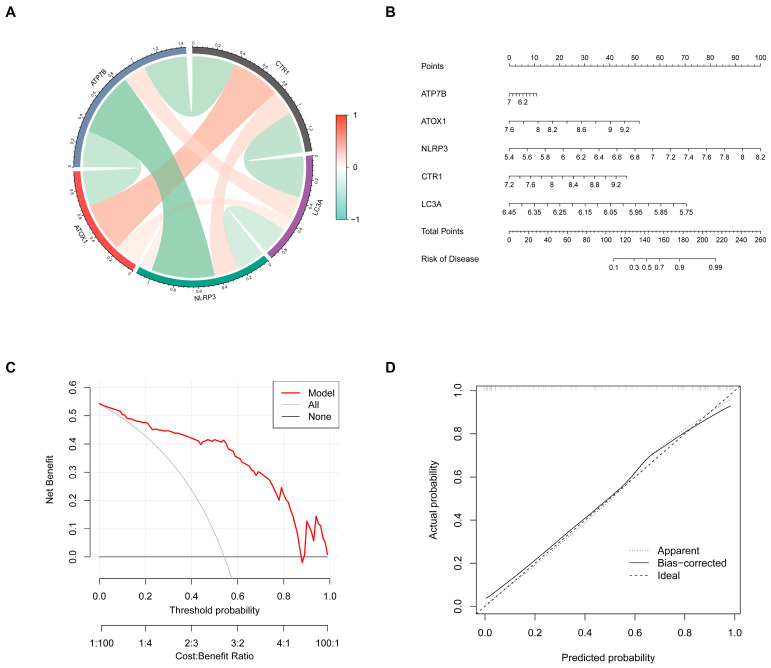
Construction of nomograph for risk prediction of unstable atherosclerotic plaque based on key genes. (**A**) The chordal graph shows the correlation between different key genes. (**B**) The nomogram shows the risk prediction model of unstable plaque based on key genes. (**C**,**D**) The decision curve and calibration curve show the prediction accuracy of the risk model.

To enhance the clinical applicability of the five-gene signature, a nomogram was constructed (Figure 8B). The nomogram integrates the expression levels of the five key genes to generate a score that predicts the risk of plaque instability. A chord diagram illustrates the significant interactions between these key genes (Figure 8A). The calibration curve demonstrates high consistency between the nomogram’s predictions and actual observations (Figure 8C). Moreover, decision curve analysis (DCA) confirmed that the nomogram provides significant clinical net benefit (Figure 8D), indicating its accuracy and reliability as a diagnostic tool.

### 3.10. Experimental Validation of Key Genes and the Functional Role of ATOX1

**Figure 9:**
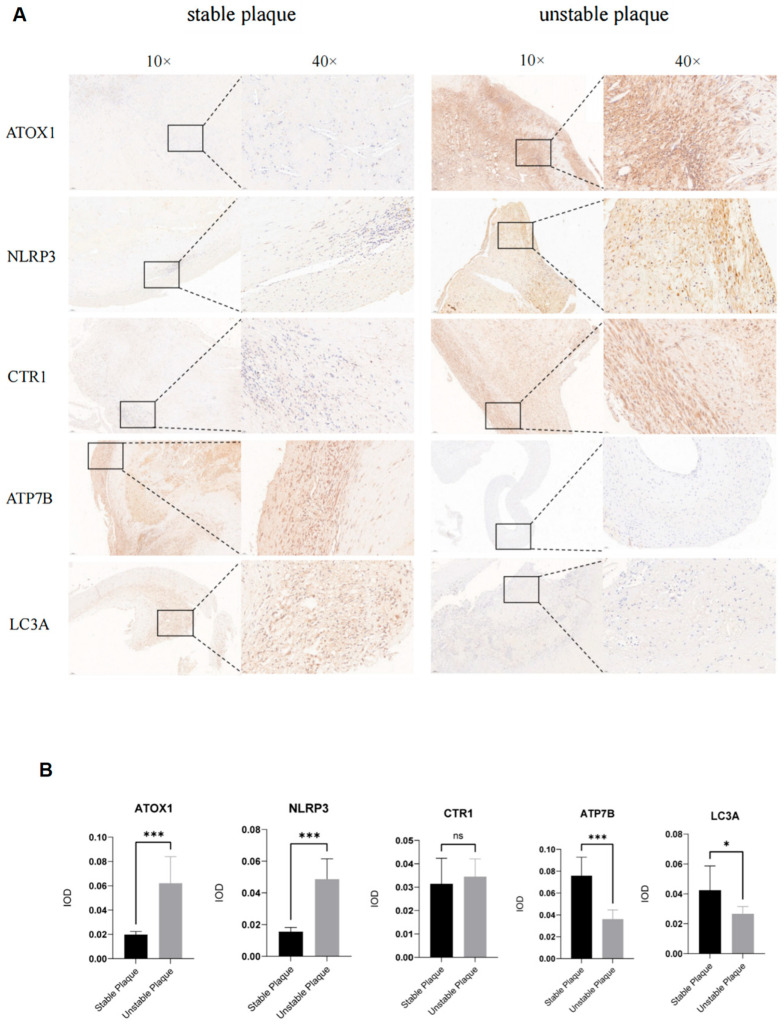
(**A**) Immunohistochemistry showed the expression of key genes ATOX1, NLRP3, CTR1, ATP7B, and LC3A in stable and unstable plaques. (**B**) The bar chart provides an objective and quantitative summary of the immunohistochemical staining. ns = not significant; * *p* < 0.05; *** *p* < 0.001. Stable plaque group *n* = 4, unstable plaque group *n* = 5; An unpaired two-tailed Student’s *t*-test was employed to compare the differences between the two groups.

First, we performed immunohistochemistry (IHC) on nine human carotid plaque samples (four stable, five unstable) (Figure 9). Consistent with our bioinformatic analysis, the expression of ATOX1 and NLRP3 was significantly up-regulated in unstable plaques, while the expression of ATP7B and LC3A was significantly down-regulated. However, the expression of CTR1 showed no significant difference between the two groups (*p* > 0.05).

**Figure 10:**
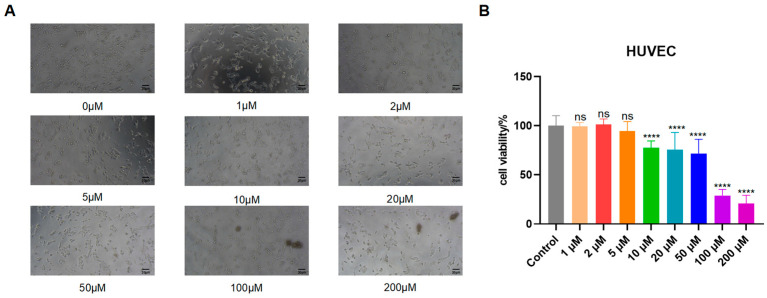
Cytotoxicity of CuSO4 in HUVEC cells. (**A**) Cells were treated with CuSO_4_ (0, 1, 2, 5, 10, 20, 50, 100, 200 μM) for 24 h, and changes in cell numbers were observed by microscopy. Scale bar 50 µm. (**B**) Cell viability was analyzed by CCK-8 assay. ns = not significant; **** *p* < 0.0001.

Second, to explore the functional impact of cuproptosis on vascular cells, we treated Human Umbilical Vein Endothelial Cells (HUVECs) with CuSO4. A CCK-8 assay showed that CuSO_4_ induced dose-dependent cell death, with significant cytotoxicity observed at concentrations ≥10 μM (Figure 10).

**Figure 11:**
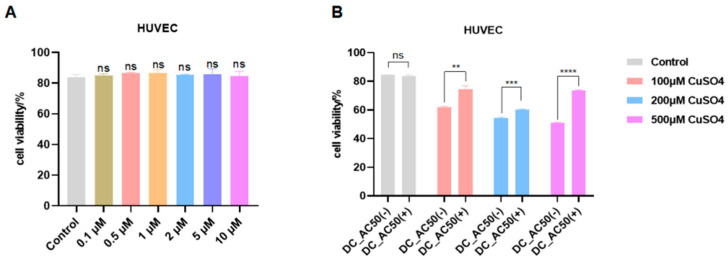
(**A**) The effect of ATOX1 inhibitors on HUVEC activity (**B**) The effect of ATOX1 inhibitor on activity of HUVECs treated with CuSO_4_. Statistical diagram. ns = not significant; ** *p* < 0.01; *** *p* < 0.001; **** *p* < 0.0001.

Third, to test the functional role of our key identified gene, ATOX1, we used the ATOX1 inhibitor DC-AC50. The inhibitor alone had no significant effect on HUVEC viability at concentrations up to 10 μM (Figure 11A). We then co-treated HUVEC with CuSO_4_ and DC-AC50. The results showed that inhibition of ATOX1 significantly rescued CuSO_4_-induced cell death (Figure 11B). Specifically, the inhibitor reduced cell death by 14.5% (*p* < 0.01) at 100 μM CuSO_4_, 5.9% (*p* < 0.001) at 200 μM CuSO_4_, and 21.8% (*p* < 0.0001) at 500 μM CuSO_4_. This provides direct functional evidence that ATOX1 is a key regulator of copper-induced death in endothelial cells.

## 4. Discussion

Recent studies have shown that cuproptosis, a newly discovered form of programmed cell death, involves all cells dominated by mitochondrial respiration. Relying on mitochondrial respiration in cells, when the concentration of copper ions in the cell exceeds the threshold, copper ions can bind to thioacyl proteins in TCA, causing abnormal oligomerization of thioacyl proteins and lowering the level of Fe-S cluster proteins. Together, the two induce protein toxicity stress reactions, ultimately leading to cell death [8]. As a newly discovered form of programmed cell death, it involves all kinds of cells that are dominated by mitochondrial respiration. Copper is reported to be closely related to the immune system [24]. Meanwhile, the occurrence of atherosclerosis is also closely related to inflammation and immunity [25,26]. However, the specific regulatory mechanism of copper-induced cell death has not been clarified, and studies on cuproptosis in cardiovascular diseases are also lacking.

In the present study, we screened 27 CRGs in atherosclerotic plaque-related datasets. GO annotation and KEGG pathway enrichment analysis showed that these CRGs were mainly enriched in copper ion homeostasis, mitochondrial matrix, mineral absorption, and the citrate cycle (TCA cycle). Our five-gene signature provides a novel mechanistic framework for plaque instability.

ATOX1, a copper chaperone, and ATP7B, a copper-exporting ATPase, are central regulators of copper homeostasis [27,28]. At the same time, ATOX1 also has an obvious antioxidant function, which can contribute to inflammatory response and protect cells against various oxidative stresses [29]. It has been demonstrated that ATOX1 is involved in vascular smooth muscle cell migration in atherosclerosis and contributes to the neointimal formation [30].

ATP7B is a copper transporter that can use copper transported by ATOX1 for incorporation into copper-dependent enzymes such as ceruloplasmin [31]. Wilson’s disease is one type of liver function injury caused by the ATP7B gene mutation, which leads to copper overload in liver cells [32]. Some studies have also found that ATP7B can impact lipid metabolism disorders [33,34]. Our in silico and IHC data revealed that unstable plaques exist in a state of profound copper dysregulation: ATOX1 is up-regulated (promoting copper uptake/transfer) while ATP7B is down-regulated (impairing copper export). This “high-influx, low-efflux” state likely leads to intracellular copper overload, the prerequisite for cuproptosis.

NLRP3 is a classic inflammasome, which can participate in the inflammatory response in atherosclerosis [35,36]. Recently, NLRP3 has been reported to cause copper-mediated neurotoxicity in animal models of Wilson disease [37]. This dual hit—copper toxicity and failed autophagy—plausibly triggers the activation of the NLRP3 inflammasome, which we also found to be significantly up-regulated.

LC3A encodes microtubule-associated proteins, which are mainly associated with autophagy. Many studies have shown that excess copper can induce autophagy and apoptosis [38,39,40]. The down-regulation of LC3A, an essential autophagy marker, suggests impaired clearance of damaged mitochondria (mitophagy), which are a known source of reactive oxygen species and damage-associated molecular patterns (DAMPs).

This allows us to propose an integrated model: ATP7B down-regulation and ATOX1 up-regulation synergistically cause copper imbalance. This leads to copper-induced cell death (targeting the TCA cycle) and, combined with impaired autophagic clearance (LC3A down-regulation), activates the NLRP3 inflammasome. The activated NLRP3 and up-regulated ATOX1 (which also has pro-inflammatory roles) then drive a feed-forward loop of inflammation and immune cell recruitment, culminating in plaque instability.

CTR1 is also a copper transporter protein widely expressed on most cells’ plasma membrane and has the same copper uptake function, playing an important role in copper homeostasis [41]. The genetic inactivation of CTR1 leads to copper deficiency in cells. The amount of copper ingested into cells depends on the abundance of CTR1 at the plasma membrane, and excessive copper can also stimulate the reticulin and kinetin-dependent endocytosis of CTR1 [42]. A key challenge, however, is the status of CTR1. While selected by all three machine learning algorithms, its protein expression was not significantly different in our IHC validation. This apparent contradiction must be addressed. First, our IHC cohort (n = 9) was small, likely lacking the statistical power to detect a subtle difference (a potential Type II error). Second, machine learning models, particularly RF and SVM, excel at capturing complex, non-linear interactions. CTR1’s importance may lie not in its independent expression level but in its combinatorial effect with the other four genes. Third, discrepancy between mRNA levels (from GEO datasets) and protein levels (from IHC) is a well-known biological phenomenon, often due to post-transcriptional regulation. Therefore, while our IHC data do not confirm CTR1 as an independent biomarker, its robust selection by machine learning suggests it remains a key component of the five-gene interaction signature, warranting further validation in larger protein cohorts.

In atherosclerosis, macrophages play a central role in the development and progression of plaque. Under different stimuli, M0 macrophage differentiated into M1 macrophage with a proinflammatory effect and M2 macrophage with an anti-inflammatory effect [43]. Studies have revealed that the instability of atherosclerotic plaques is related to the degree of inflammation [44]. A major finding of this study is the significant infiltration of M0 macrophages in unstable plaques, coupled with a reduction in M2 macrophages. This deviates from the classic M1/M2 paradigm, which would predict high M1 infiltration. As suggested in the original manuscript, this high M0 population may represent a “pro-inflammatory reservoir” of newly recruited monocytes whose polarization to a mature M1 or M2 phenotype is stalled or dysregulated by the plaque’s toxic microenvironment. This high M0 infiltration itself, rather than M1, may thus be a key indicator of instability.

To better understand the biological function of these key genes and their relationship with immunity, we constructed two subtypes of these datasets based on the expression of the five genes and conducted pathway enrichment analysis and immune cell infiltration analysis on this basis. In the two subtypes, ATOX1, NLRP3, and CTR1 were highly expressed in the C1 group, and ATP7B was highly expressed in the C2 group. The results showed that, compared with C1, the C2 subtype significantly up-regulated gene expression in the “Toll-like receptor signaling pathway,” “Nod-like receptor signal pathway,” “Sphingolipid metabolism,” and “PPAR signaling pathway.”

In the innate immune system, innate immune cells recognize exogenous pathogen-associated molecular patterns (PAMPs) and endogenous damage-associated molecular patterns (DAMPs) through pattern recognition receptors (PRRs). Both toll-like and nod-like receptors are PRRs, but Toll-like receptors are located on the cell membrane to recognize ligands in the extracellular environment. In contrast, Nod-like receptors are located in the cytoplasm to recognize ligands in the intracellular environment. Evidence is accumulating that TLRs play a key role in the occurrence and development of atherosclerosis [45,46]. The most representative Nod-like receptor is the inflammasome NLRP3, which mediates pyroptosis through the activation of caspase-1. It has been reported that inhibition of NLRP3 activation can prevent the progression of atherosclerosis by reducing macrophage inflammation and pyroptosis [47,48]. Sphingolipids play an important role in cell membrane formation, signal transduction, and plasma lipoprotein metabolism. The impairment of sphingolipid metabolism can lead to lipid deposition in the vessel wall and aggravate the progression of atherosclerosis [49,50]. The peroxisome proliferator-activated receptors (PPARs), including PPAR-α, PPAR-β/δ, and PPAR-γ (a nuclear receptor activated by fatty acids and their derivatives), play crucial roles in glucose and lipid metabolism [51,52]. For atherosclerosis, the PPARs are considered important therapeutic targets [53,54].

Our subtype analysis provides a compelling explanation for this phenomenon. We found that this high M0 infiltration is not universal but is primarily a feature of the C1 subtype. This C1 subtype is, in turn, defined by high expression of ATOX1 and NLRP3, both of which were strongly correlated with M0 infiltration. This suggests a specific destabilization pathway, i.e., C1 (ATOX1/NLRP3-driven), characterized by copper dysregulation, inflammasome activation, and a stalled M0 macrophage phenotype.

In stark contrast, the C2 subtype exhibited a completely different profile. It was characterized by high ATP7B expression and significant enrichment of broad inflammatory pathways, including Toll-like receptor and Nod-like receptor signaling. Its immune profile was “immune-hot,” with high infiltration of M1 macrophages and CD8 T cells. This suggests a second, independent destabilization pathway: C2 (TLR/broad inflammation-driven), which may be less dependent on cuproptosis and more related to classic, broad-spectrum innate immune activation.

This C1/C2 dichotomy is perhaps our most novel finding, suggesting that “plaque instability” is mechanistically heterogeneous. This has significant clinical implications: patients with the C1 (NLRP3-driven) subtype might be ideal candidates for targeted IL-1β therapies (e.g., Canakinumab, from the CANTOS trial), whereas C2 patients may require broader anti-inflammatory strategies.

Bioinformatic analyses, while powerful, generate correlations and hypotheses that require experimental validation. A major strength of our study is the functional validation of ATOX1, a key gene identified in our five-gene signature and confirmed by IHC to be up-regulated in unstable plaques. We demonstrated that copper sulfate induces dose-dependent death in HUVECs, a critical cell type lining the vascular wall. More importantly, we showed that specific inhibition of ATOX1 using DC-AC50 significantly rescued HUVECs from this copper-induced death, reducing cell death by up to 21.8% (*p* < 0.0001). This finding is critical as it moves ATOX1 from a simple biomarker to a functionally implicated gene. It provides the first direct evidence that ATOX1, previously known for its role in vascular smooth muscle cell migration, is also a direct modulator of cuproptosis in endothelial cells. This strongly supports our central hypothesis and suggests that targeting the ATOX1-mediated copper homeostasis pathway could be a novel therapeutic strategy for stabilizing atherosclerotic plaques.

Regarding potential crosstalk with ferroptosis, while our study focuses on cuproptosis, it is worth noting that the identified key genes may also contribute to plaque instability through ferroptosis. Copper homeostasis is intrinsically linked to iron metabolism [55]. ATP7B is required for the metallation of ceruloplasmin, a ferroxidase essential for iron efflux. The down-regulation of ATP7B observed in unstable plaques could impair ceruloplasmin activity, leading to intracellular iron accumulation and subsequent ferroptosis [56]. Furthermore, LC3A-mediated autophagy can regulate cellular iron levels through ‘ferritinophagy’ (the autophagic degradation of ferritin). The dysregulation of LC3A may disrupt this balance, sensitizing cells to lipid peroxidation [56]. Finally, the NLRP3 inflammasome, which we found to be up-regulated, is a known downstream effector activated by ferroptotic cell death [57]. Therefore, the gene signature identified here likely reflects a broader disruption of metal ion homeostasis, where cuproptosis and ferroptosis may synergistically drive plaque necrosis and instability.

## 5. Conclusions

Our study provides novel bioinformatic and experimental evidence that cuproptosis-related genes (CRGs) are intrinsically linked to the instability of atherosclerotic plaques. We identified and validated a robust five-gene signature (LC3A, ATP7B, ATOX1, CTR1, NLRP3) capable of diagnosing unstable plaques. More importantly, we discovered that unstable plaques are heterogeneous, comprising at least two molecular subtypes (C1 and C2) with distinct immune profiles driven by these CRGs. Finally, we provided the first functional evidence that the key signature gene ATOX1 is a direct regulator of copper-induced endothelial cell death. These findings not only deepen the understanding of copper metabolism in atherosclerosis but also identify the cuproptosis pathway, and ATOX1 in particular, as a promising new target for future plaque stabilization therapies.

## 6. Limitations and Future Directions

Despite these novel findings, our study has several limitations that must be acknowledged. First, the bioinformatic analysis relies on public GEO datasets, which have inherent heterogeneity. Furthermore, CIBERSORT is an in silico deconvolution algorithm, and its immune cell estimations require validation by more direct methods like flow cytometry or single-cell RNA sequencing. Second, our experimental validations are preliminary. The IHC cohort was small (n = 9), which, as discussed, limits the conclusions that can be drawn about CTR1. Third, our functional validation was primarily conducted in Human Umbilical Vein Endothelial Cells (HUVECs). While endothelial injury is a critical initiating factor in atherosclerosis, the plaque environment is complex. Our IHC results (Figure 9) show that key genes like ATOX1 are expressed throughout the plaque tissue, suggesting their potential involvement in other cell types such as vascular smooth muscle cells (VSMCs) and macrophages. Since we did not perform in vitro experiments on VSMCs or macrophages, the specific interactions between ATOX1 and CTR1 or other cuproptosis regulators in these cell populations remain to be fully elucidated. Future studies using co-culture systems or cell-specific knockout models are needed to address this limitation.

Future research must focus on validating these findings in in in vivo animal models, such as ApoE-/- or Ldlr-/- mice. It will be crucial to determine if the five-gene signature can track plaque progression in these models and whether the C1/C2 subtypes are discernible. Most importantly, the therapeutic potential of ATOX1 inhibition (via DC-AC50) must be tested in vivo to assess its ability to reduce copper accumulation, decrease inflammation, and promote plaque stability.

## Data Availability

The data that support the findings of this study are openly available in GEO database at https://www.ncbi.nlm.nih.gov/ (accessed on 15 December 2023).

## Supplementary Materials

The following supporting information can be downloaded at: https://www.mdpi.com/article/10.3390/biomedicines13122983/s1, Figure S1: the immune factors affecting the instability of atherosclerotic plaque.
