# Supplementary material for: Analysis of the Correlation Between Cuproptosis and Instability of Atherosclerotic Plaques
Source: Biomedicines. 2025 Dec 4;13(12):2983. doi: 10.3390/biomedicines13122983 (PMC12730235; doi:10.3390/biomedicines13122983)
Supplement: Supplementary file 1 [file biomedicines-13-02983-s001.zip › biomedicines-3935300-supplementary.pdf]

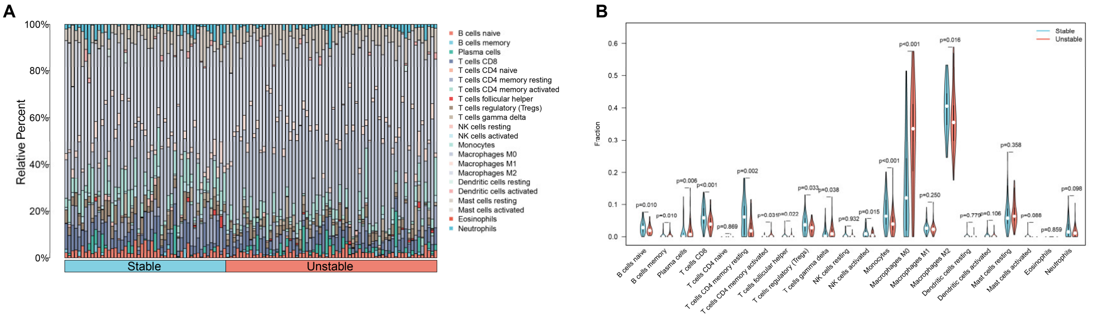

Figure S1. the immune factors affecting the instability of atherosclerotic plaque. (A) The histogram showed the infiltration rate of 22 immune cells in the samples of stable plaque and unstable plaque. (B) the violin plot showed the differences in the infiltration of immune cells between the two groups.
